# Supplementary material for: Evolution of thermal performance curves: A meta‐analysis of selection experiments
Source: J Evol Biol. 2022 Sep 21;36(1):15–28. doi: 10.1111/jeb.14087 (PMC10087336; doi:10.1111/jeb.14087)
Supplement: Supplementary file 1 — Appendix S1 [file JEB-36-15-s001.pdf]

Supplementary Material

Sarthak P. Malusare, Giacomo Zilio and Emanuel A. Fronhofer:

Evolution of thermal performance curves: a meta-analysis of selection experiments

S1 Web of Science search algorithm

ts = (temperature OR thermal)

AND

ts = (experimental evolution OR artificial selection OR selection experiment OR laboratory selection OR adaptation)

AND

ts = (microcosm* OR mesocosm* OR chemostat* OR experiment* OR evolutionary rescue)

NOT

ts=(cancer* OR cardio* OR surg* OR carcin* OR medic* OR drug*)

AND

wc = (Behavioral Sciences OR Ecology OR Evolutionary Biology OR Limnology OR Marine & Freshwater Biology OR Microbiology OR Multidisciplinary Sciences OR Plant Sciences OR Zoology OR Biochemical Research Methods OR Environmental Sciences OR Genetics & Heredity OR Biochemistry & Molecular Biology OR Life Sciences & Biomedicine - Other Topics)

AND

py = 1900-2021

ISI WoS field tag explanation: ts (Topic), wc (Web of Science Categories), py (Year Published)

S2 Criteria for rejection of papers

1. Ecological papers with transplant experiments, i.e., no selection experiment or experimental evolution performed.

2. Ecological papers measuring the TPC without a selection experiment.

3. Ecological papers in which study organisms have to actively select a thermal environment in a choice experiment, e.g. along a thermal gradient arena, in order to study for example, matching habitat choice.

4. Ecological papers with parental effects of temperature treatment on the offspring and their response which are generally measured over only one generation.

5. Experimental evolution papers with only life-history traits like body size, developmental time, and no direct fitness measure reported.

6. Experimental evolution papers with a direct fitness measure present but the assay temperatures for the selection and the control/ ancestor lines do not correspond making it impossible to calculate relative fitness at a particular assay temperature.

7. Experimental evolution experiments where a control or an ancestor are absent and only the fitness trajectory of the selection line is studied with increasing temperature.

8. Experimental evolution experiments that did not report standard errors or other error estimates.

S3 Supplementary statistical methods

Direct response to selection

We fitted linear mixed models to this data subset comprising all additive models combining the following explanatory variables: relative selection temperature as a categorical explanatory variable (higher, lower or equal compared to the control treatment) and type of genetic variation used (standing genetic variation vs. de novo variation based on mutations). This analysis included 230 data points from 40 studies and 28 species.

We chose vaguely informative priors: the intercept parameter priors for all models followed a normal distribution with mean 0 and standard deviation 2. The mean and standard deviation of the random effect hyperpriors followed a normal distribution with mean 0 and standard deviation 3.

We further ran a supplementary analysis using the same basic model set but adding the number of generations an experiment lasted as another explanatory variable. For this analysis, we standardised the number of generations to avoid divergent transitions and removed four studies (seven data points) that did not report the number of generations (normal prior mean 0 and standard deviation 1). The additional analysis included 223 data points from 36 studies and 26 species.

Two-point analysis

In analogy to the analysis of the direct response to selection, we fitted linear mixed models explaining relative fitness as a function of (absolute) relative assay temperature. In the full model, we allowed for different slopes and intercepts depending on whether experimental populations had evolved at higher or lower temperature compared to the control and tested in the final assay at high or low temperature. We subsequently included an additional explanatory variable, the type of genetic variation, as varying intercept, slope, both intercept and slope. The intercept and slope parameter priors for all models followed a normal distribution with mean 0 and standard deviation 1. The mean and standard deviation of the random effect hyperpriors followed a normal distribution with mean 0 and standard deviation 1. This analysis included 122 data points in 61 TPCs from 18 studies and 15 species.

A supplementary analysis was conducted using the same model details but adding the standardized number of generations as additional explanatory variable. Due to missing data on the number of generations, we removed 10 TPCs from this additional analysis. This analysis included 102 data points in 51 TPCs from 13 studies and 12 species.

Multipoint analysis

Here, we first fitted mixed models including intercept-only, linear, quadratic and cubic models of relative fitness as a function of standardized relative assay temperature. Note that these shapes are descriptive and flexible enough to describe the shapes we find. We do not imply that TPCs are actually cubic. All priors followed a normal distribution with mean 0 and standard deviation 1. The mean and standard deviation of the random effect hyperpriors followed a normal distribution with mean 0 and standard deviation 1. This analysis included 600 data points in 159 TPCs from 21 studies and 13 species.

We also added the type of genetic variation as an explanatory variable. We fitted supplementary models with the inclusion of the number of generations (standardized) as an extra explanatory variable. Due to missing data on the number of generations, we removed 3 TPCs from this additional analysis. This analysis included 586 data points in 156 TPCs from 19 studies and 11 species.

"Hotter is better"

Similar to the direct response to selection analysis (see above), we fitted two linear mixed models: an intercept model to test whether the difference in maximal relative fitness was different from zero and a second one in which the temperature selection regime (selection at higher or lower temperatures than the ancestor/ control) was added as a categorical fixed effect. In both models, the priors for the intercepts followed a normal distribution with mean 0 and standard deviation 2. The random effect hyperprior mean and standard deviation followed a normal distribution with mean 0 and standard deviation 3. This analysis included 27 data points from 11 studies and 9 species.

We then added to this model the extra explanatory variable type of genetic variation as varying intercept. We conducted a supplementary analysis further considering number of generations (standardized) as varying slope. Here, we removed one study not reporting this information. This analysis included 26 data points from 10 studies and 8 species.

Assessment of publication bias

We assessed publication bias using funnel plots (Sterne *et al.*, 2001; Sterne & Egger, 2001; O'Dea *et al.*, 2021), where the inverse of the standard error is plotted against relative fitness. In the absence of bias, funnel plots are expected to be symmetrical. In addition to visual inspection, symmetry can be inspected by means of linear regression of the two variables. The regression line is expected to intercept the x-axis close to the origin if there is bias. Alternatively, if the studies are evenly distributed and thus there is no bias towards high or low relative fitness values, a zero-slope (horizontally flat line) may be expected.

We inspected the symmetry not only visually, but we also tested and quantified it by fitting an

intercept model and a linear regression model (chain length: warmup = 1,000 iterations, chain = 30,000 iterations). The intercept and slope priors for both models followed a normal distribution with mean 0 and standard deviation 1. Relative fitness was standardised in cases of divergent transitions. We included species ID, study ID and TPC ID as random effects as in the main analyses. The mean and standard deviation of the random effect hyperpriors followed a normal distribution with mean 0 and standard deviation 1. Based on WAIC weights we averaged the posterior predictions of the models.

S4 Supplementary figures

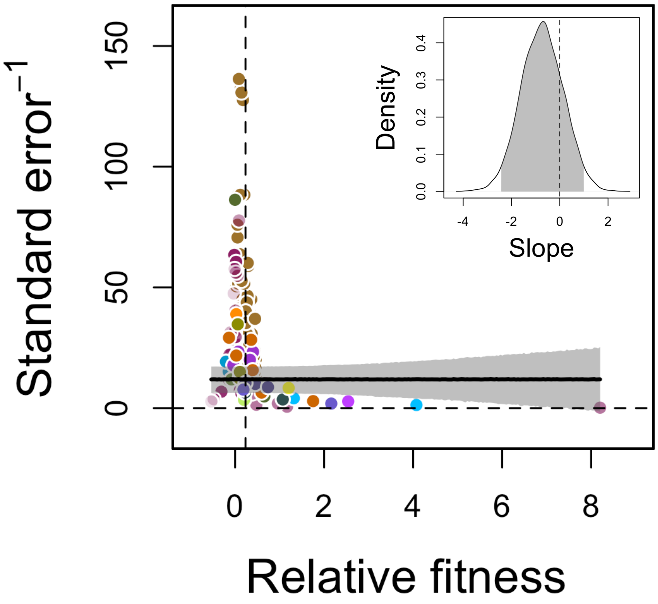

Figure S1: Funnel plot of the analysis of assay data measured at the same temperature experienced during experimental evolution. Each point is a study with different colours per species (color code see Fig. 3). The shaded area and thick line are the overall 95% compatibility interval and median of the averaged posterior predictions based on WAIC weights. Model selection results are reported in Table S3. The random effect structure used for the corresponding main analysis was included here. The insert panel highlights the posterior distribution of the slope parameter which clearly overlaps zero.

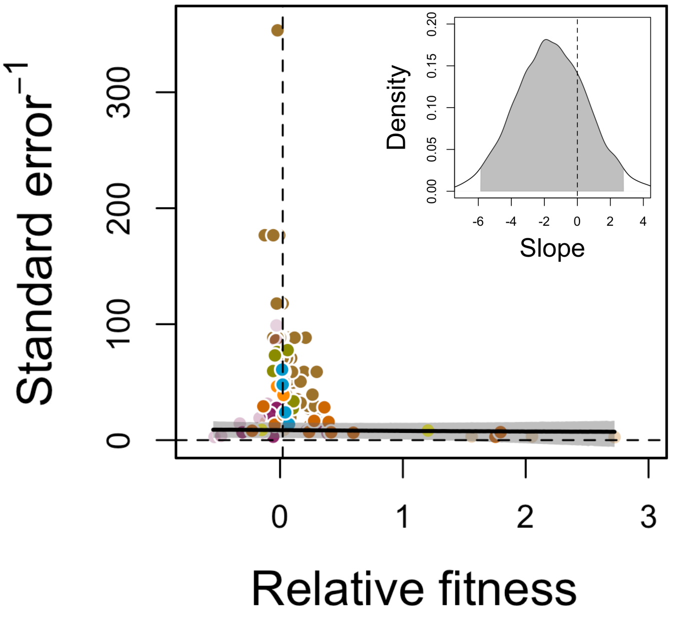

Figure S2: Funnel plot of the two temperature assay point analysis. Each point is a study with different colours per species (color code see Fig. 3). The shaded area and thick line are the overall 95% compatibility interval and median of the averaged posterior predictions based on WAIC weights. Model selection results are reported in Table S4. The random effect structure used for the corresponding main analysis was included here. The insert panel highlights the posterior distribution of the slope parameter which clearly overlaps zero.

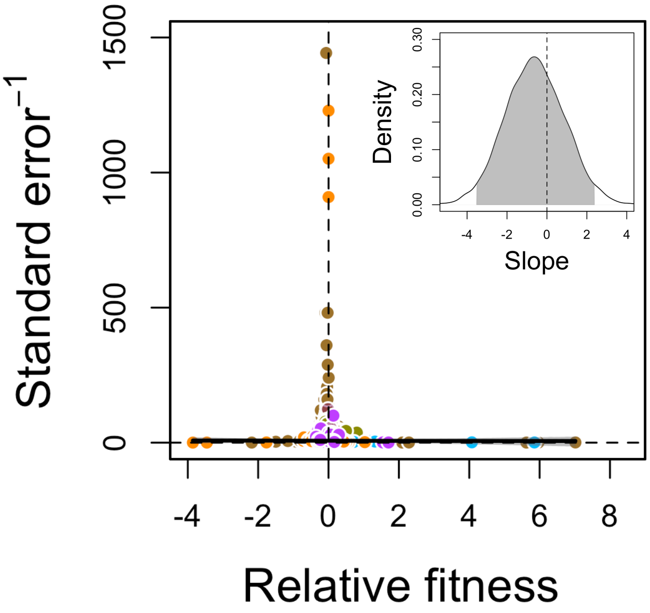

Figure S3: Funnel plot of the multipoint temperature assay analysis. Each point is a study with different colours per species (color code see Fig. 3). The shaded area and thick line are the overall 95% compatibility interval and median of the averaged posterior predictions based on WAIC weights. Model selection results are reported in Table S5. The random effect structure used for the corresponding main analysis was included here. The insert panel highlights the posterior distribution of the slope parameter which clearly overlaps zero.

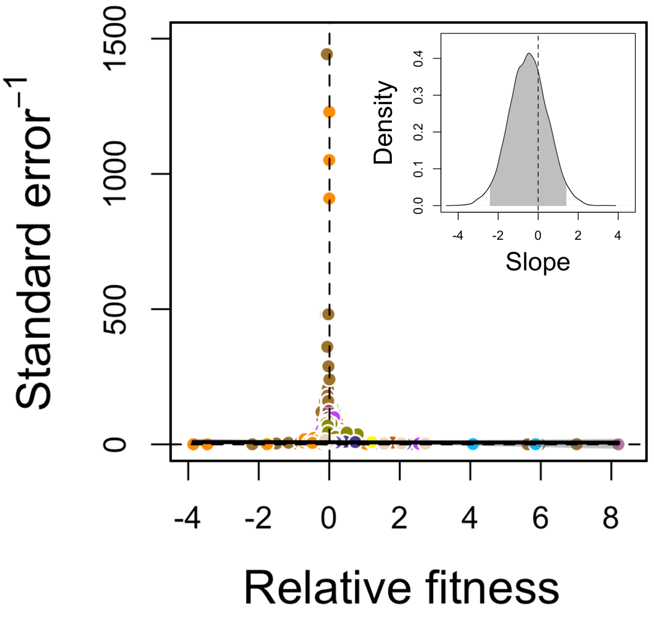

Figure S4: Funnel plot of all data together, including one, two, and multipoint temperature assay analysis. Each point is a study with different colours per species (color code see Fig. 3). The shaded area and thick line are the overall 95% compatibility interval and median of the averaged posterior predictions based on WAIC weights. Model selection results are reported in Table S6. The random effect structure used for the corresponding main analysis was included here. The insert panel highlights the posterior distribution of the slope parameter which clearly overlaps zero.

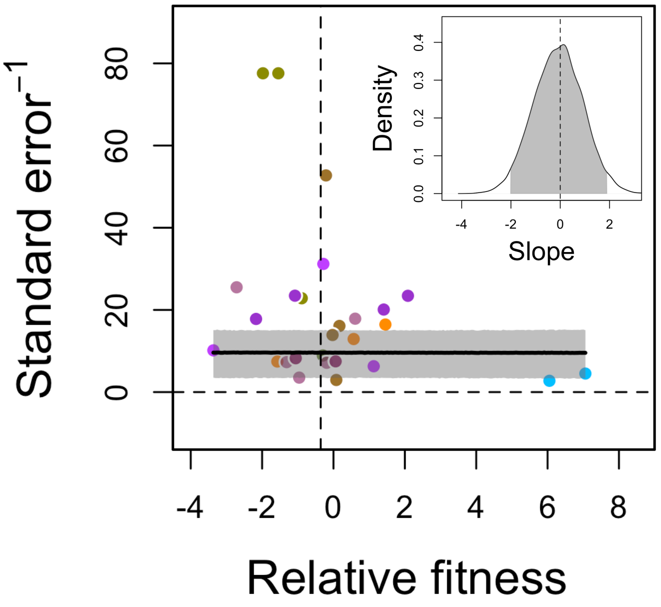

Figure S5: Funnel plot of the "Hotter is better" analysis. Each point is a study with different colours per species (color code see Fig. 3). The shaded area and thick line are the overall 95% compatibility interval and median of the averaged posterior predictions based on WAIC weights. Model selection results are reported in Table S7. The random effect structure used for the corresponding main analysis was included here. The insert panel highlights the posterior distribution of the slope parameter which clearly overlaps zero.

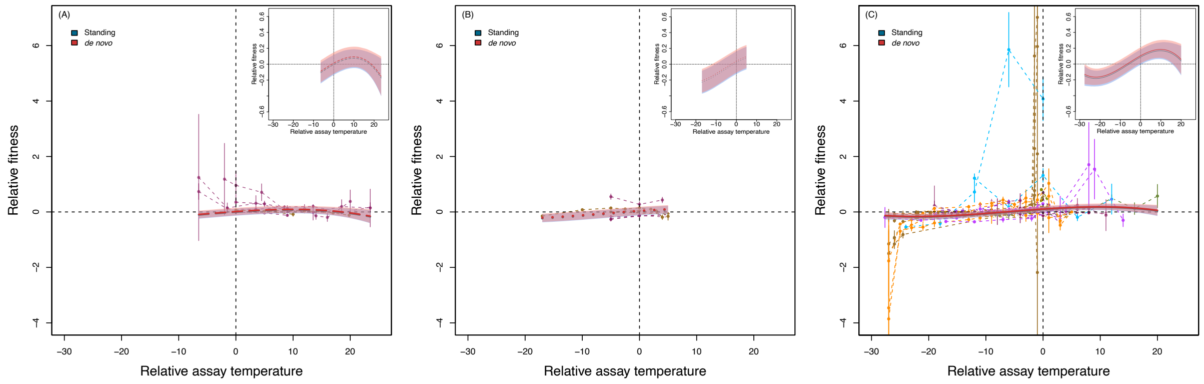

Figure S6: Evolution of the TPC. The plot shows relative fitness as a function of relative assay temperature, that is, the difference between selection and assay temperature, for studies that assayed selected lines at three and more temperatures allowing us to infer changes in TPC shape. Each combination of points connected by a dashed line corresponds to one TPC and the error bars represent the associated standard error. Different colours correspond to different species as shown in Fig. 3. The solid lines visualize the median model predictions and the shaded areas are the 95% compatibility interval. Here, the best model (Tables S12 and S13) is a cubic model model that includes an effect of the type of genetic variation used in the experiments (standing genetic variation vs. de novo mutations; represented in blue and red, respectively) and relative selection temperature ('Sign'), that is, whether selection happened at lower (A), equal (B) or higher (C) temperatures compared to the control treatment. The insets show only the statistical model prediction for better visibility of the compatibility intervals.

S5 Supplementary tables

Table S1: Overview of selected papers, with taxonomic information, fitness measure used, source of data, as well as details on the calculation of relative fitness which is described in the main text. Equation numbers refer to Chevin (2011).

**Reference**	**Species**	**Fitness measure**	**Data source**	**Relative fitness calculation**
Cullum *et al.* (2001)	*Escherichia coli*	Relative fitness	Paper	Eq. 3.2
Dam *et al.* (2021)	*Acartia tonsa*	Population fitness	Graph	Eq. 3.2
Barton *et al.* (2020)	*Ostreococcus tauri*	Growth rate	SI	Eq. 3.2
Frenck *et al.* (2013)	*Brassica napus*	Seed quantity	Graph	Eq. 2.5
Gomez-Llano *et al.* (2021)	*Drosophila melanogaster*	Number of offspring produced	Graph	Eq. 2.5
Rouco *et al.* (2011)	*Microcystis aeruginosa*	Doubling time	Graph	Eq. 3.2
Sandberg *et al.* (2014)	*Escherichia coli*	Population growth rate	Paper	Eq. 3.2
Wu *et al.* (2020)	*Phytophthora infestans*	Colony size	SI	Eq. 2.5
Bennett & Lenski (2007)	*Escherichia coli*	Relative fitness	Paper	Eq. 3.2
Zhang *et al.* (2019)	*Daphnia magna*	Intrinsic rate of increase	SI	Eq. 2.5
Chakravarti *et al.* (2017)	*Symbiodinium* sp.	Specific growth rate	SI	Eq. 3.2
Duncan *et al.* (2011)	*Paramecium caudatum*	Area under the growth curve	Author	Eq. 3.2
Van Doorslaer *et al.* (2009)	*Daphnia magna*	Performance	Graph	Eq. 2.5
Plesnar-Bielak *et al.* (2012)	*Rhizoglyphus robini*	Fecundity	Graph	Eq. 2.5
Partridge *et al.* (1995)	*Drosophila melanogaster*	Fecundity	Paper	Eq. 2.5
Tseng & O'Connor (2015)	*Daphnia pulex*	Per capita growth rate	Graph	Eq. 2.5
Hallsson & Björklund (2012)	*Callosobruchus maculatus*	Fecundity	Graph	Eq. 2.5
Shi & Xia (2003)	*Pseudomonas pseudoalcaligenes*	Maximum growth rate	Paper	Eq. 3.2
Batarseh *et al.* (2020)	*Escherichia coli*	Relative fitness	SI	Eq. 3.2
Koch & Guillaume (2020)	*Tribolium castaneum*	Offspring number	Graph	Eq. 2.5
Lind *et al.* (2020)	*Caenorhabditis remanei*	Daily growth factor	Graph	Eq. 2.5
Schluter *et al.* (2014)	*Emiliania huxleyi*	Exponential growth rate	Graph	Eq. 3.2
Tarkington & Zufall (2021)	*Tetrahymena thermophila*	Relative fitness	Paper	Eq. 3.2
Van Doorslaer *et al.* (2010)	*Daphnia magna*	Growth rate	Graph	Eq. 2.5
Breckels *et al.* (2014)	*Poecilia reticulata*	Brood size	Paper	Eq. 2.5
Xu (2004)	*Cryptococcus neoformans*	Relative fitness colony size	Paper	Eq. 2.5
Bennett *et al.* (1992)	*Escherichia coli*	Relative fitness	Paper data	Eq. 3.2
Santos (2007)	*Drosophila subobscura*	Average relative fitness	Graph	Eq. 2.5
Van Doorslaer *et al.* (2007)	*Simocephalus vetulus*	Performance	Graph	Eq. 3.2
Berger *et al.* (2014)	*Sepsis punctum*	Growth rate	SI	Eq. 2.5
Rodriguez-Verdugo *et al.* (2014)	*Escherichia coli*	Relative fitness	SI	Eq. 3.2
Fragata *et al.* (2016)	*Drosophila subobscura*	Fecundity	SI	Eq. 2.5
Pfenninger & Foucault (2020)	*Chironomus riparius*	Population growth rate	Author	Eq. 2.5
Santos *et al.* (2006)	*Drosophila subobscura*	Viability	Graph	Eq. 2.5
Schaum *et al.* (2017)	*Chlamydomonas reinhardtii*	ln mu_max	Graph	Eq. 3.2
Cooper *et al.* (2001)	*Escherichia coli*	Maximum growth rate	Graph	Eq. 3.2
Mongold *et al.* (1996)	*Escherichia coli*	Relative fitness	Graph	Eq. 3.2
Killeen *et al.* (2017)	*Paramecium caudatum*	Growth rate	SI	Eq. 3.2
O'Donnell *et al.* (2018)	*Thalassiosira pseudonana*	Specific growth rate	SI	Eq. 3.2
Gilchrist *et al.* (1997)	*Drosophila melanogaster*	Walking speed	Graph	Eq. 2.5
Listmann *et al.* (2016)	*Emiliania huxleyi*	Exponential growth rate	SI	Eq. 3.1
Tseng *et al.* (2019)	*Daphnia pulex*	Per capita growth rate	SI	Eq. 2.5
Aranguren-Gassis *et al.* (2019)	*Chaetoceros simplex*	Growth rate	SI	Eq. 3.1
Condon *et al.* (2014)	*Drosophila melanogaster*	Fecundity	Graph	Eq. 2.5
Romero-Olivares *et al.* (2015)	*Neurospora discreta*	Mycelial growth rate	Graph	Eq. 2.5
Schaum *et al.* (2018)	*Thalassiosira pseudonana*	ln(growth rate)	Graph	Eq. 3.2
Mongold *et al.* (1999)	*Escherichia coli*	Absolute fitness	Graph	Eq. 3.1

Table S2: Number of studies and the number of TPCs associated with each organism for all selected studies of the meta-analysis.

**Species**	**No. of studies**	**No. of TPCs**
Daphnia magna	3	5
Daphnia pulex	3	4
Simocephalus vetulus	1	2
Acartia tonsa	1	3
Drosophila subobscura	3	8
Drosophila melanogaster	4	9
Sepsis punctum	1	1
Chironomus riparius	1	2
Tribolium castaneum	1	1
Callosobruchus maculatus	1	1
Rhizoglyphus robini	1	2
Caenorhabditis remanei	1	2
Poecilia reticulata	1	1
Cryptococcus neoformans	1	2
Neurospora discreta	1	2
Ostreococcus tauri	1	2
Chlamydomonas reinhardtii	1	1
Brassica napus	1	1
Chaetoceros simplex	1	4
Thalassiosira pseudonana	3	5
Phytophthora infestans	1	2
Tetrahymena thermophila	1	6
Paramecium caudatum	2	4
Symbiodinium sp.	1	1
Emiliania huxleyi	2	6
Microcystis aeruginosa	1	3
Synechococcus sp.	1	2
Pseudomonas pseudoalcaligenes	1	3
Escherichia coli	9	164

Table S3: Model selection table for analysis of the overall effect of selection funnel plot (fitness data measured at the same temperature as experienced during selection). We report WAIC, the standard error of WAIC ('SE'), the WAIC different to the bets model (dWAIC) as well as the WAIC weights.

**Model**	**WAIC**	**SE**	**dWAIC**	**weight**
intercept	1940.3	37.52	0	0.59
intercept + slope	1941.0	37.52	0.7	0.41

Table S4: Model selection table for two-point analysis funnel plot. We report WAIC, the standard error of WAIC ('SE'), the WAIC different to the bets model (dWAIC) as well as the WAIC weights.

**Model**	**WAIC**	**SE**	**dWAIC**	**weight**
intercept + slope	1287.9	65.56	0.0	0.56
intercept	1288.4	65.37	0.5	0.44

Table S5: Model selection table for multi-point analysis funnel plot. We report WAIC, the standard error of WAIC ('SE'), the WAIC different to the bets model (dWAIC) as well as the WAIC weights.

**Model**	**WAIC**	**SE**	**dWAIC**	**weight**
intercept + slope	7534.4	284.39	0.0	0.53
intercept	7534.6	284.38	0.2	0.47

Table S6: Model selection table for the funnel plot representing all data pooled. We report WAIC, the standard error of WAIC ('SE'), the WAIC different to the bets model (dWAIC) as well as the WAIC weights.

**Model**	**WAIC**	**SE**	**dWAIC**	**weight**
intercept	9278.1	328.62	0.0	0.52
intercept + slope	9278.3	329.05	0.2	0.48

Table S7: Model selection table for the "Hotter is better" analysis. We report WAIC, the standard error of WAIC ('SE'), the WAIC different to the bets model (dWAIC) as well as the WAIC weights.

**Model**	**WAIC**	**SE**	**dWAIC**	**weight**
intercept	250.4	23.92	0.0	0.51
intercept + slope	250.4	23.96	0.1	0.49

Table S8: Model selection table for the overall response to thermal selection. Explanatory variables are 'Type of genetic variation' (standing genetic variation vs. de novo variation based on mutations; 'Var') and 'Relative selection temperature' (higher, lower or equal compared to the control treatment; 'Sign'). In the table '+' indicates an additive effect. We report WAIC, the standard error of WAIC ('SE'), the WAIC different to the bets model (dWAIC) as well as the WAIC weights.

**Model**	**WAIC**	**SE**	**dWAIC**	**weight**
Var	1623663799	485755802	0	1
Var + Sign	1714151640	513056393	90487841	0
Intercept	1782783610	534129409	159119811	0
Sign	1930577420	578697354	306913621	0

Table S9: Model selection table for the overall response to thermal selection using the reduced data set to included number of generations in the analysis. Explanatory variables are 'Type of genetic variation' (standing genetic variation vs. de novo variation based on mutations; 'Var'), 'Relative selection temperature' (higher, lower or equal compared to the control treatment; 'Sign') and number of generations in the experiment ('Gen'; standardized). In the table '+' indicates an additive effect and ':' an interactive effect. We report WAIC, the standard error of WAIC ('SE'), the WAIC different to the bets model (dWAIC) as well as the WAIC weights.

**Model**	**WAIC**	**SE**	**dWAIC**	**weight**
Var	1711459014	523308177	0	1
Gen + Var:Gen	1737972889	531378488	26513874	0
Var + Gen	1741041679	532422388	29582665	0
Intercept	1776068393	543640762	64609379	0
Var + Gen + Var:Gen	1792392991	548092410	80933976	0
Var + Sign	1819269616	556466483	107810601	0
Var + Gen + Var:Gen + Sign	1844078940	564070080	132619926	0
Gen	1858258633	568885469	146799619	0
Gen + Var:Gen + Sign	1861953281	569617448	150494267	0
Sign	1886799538	577675125	175340524	0
Var + Gen + Sign	1895395580	579802412	183936565	0
Sign + Gen	1925653718	589701442	214194704	0

Table S10: Model selection table for the two-point response to thermal selection analysis. Explanatory variables are absolute relative assay temperature (abbreviated as 'Temp' in the table), sign of the assay temperature, that is, whether the second assay was carried out above, at, or below the selection temperature (abbreviated as 'Sign' in the table) and type of genetic variation ('Var'). Note that in the analysed studies 'Sign' correlates with the temperature of the assay: studies that performed selection at the same or lower temperatures in comparison to the control always performed the second assay at higher temperatures compared to the first assay point, for studies selecting at higher temperatures than the control the assay was always done at temperatures below the first assay point. In the table '+' indicates an additive effect and ':' an interactive effect. We report WAIC, the standard error of WAIC ('SE'), the WAIC different to the bets model (dWAIC) as well as the WAIC weights.

**Model**	**WAIC**	**SE**	**dWAIC**	**weight**
Intercept	23614381787	15324934662	0	1
Temp + Var:Temp	23717825649	15392349383	103443862	0
Temp	23757133779	15417003258	142751992	0
Var	24145184487	15671246974	530802700	0
Temp + Var	24194571950	15702742522	580190163	0
Sign	24558076629	15939269068	943694842	0
Var + Sign	24590169935	15961464453	975788148	0
Temp + Sign	24724264320	16046580988	1109882532	0
Temp + Var + Var:Temp	24884526473	16152985021	1270144686	0
Temp + Sign + Var:Temp	25003952581	16229373382	1389570794	0
Temp + Var + Var:Temp + Sign	25275487420	16406777226	1661105632	0
Temp + Var + Sign	25543644950	16580953259	1929263162	0

Table S11: Model selection table for the two-point response to thermal selection analysis with additional explanatory variables including number of generations (reduced data set). Explanatory variables are absolute relative assay temperature (abbreviated as 'Temp' in the table), Type of genetic variation ('Var'), sign of the assay temperature, that is, whether the second assay was carried out above, at, or below the selection temperature (abbreviated as 'Sign' in the table) and the number of generations the experiment lasted ('Gen'; standardized). In the table '+' indicates an additive effect and ':' an interactive effect. We report WAIC, the standard error of WAIC ('SE'), the WAIC different to the bets model (dWAIC) as well as the WAIC weights.

**Model**	**WAIC**	**SE**	**dWAIC**	**weight**
Intercept	6354484583	4121821808	0	1
Gen	6391495482	4144758709	37010899	0
Gen + Temp	6394900591	4146133403	40416008	0
Temp	6401329257	4151651200	46844674	0
Temp + Temp:Var	6445826040	4180753202	91341457	0
Sign	6466283750	4194914902	111799167	0
Gen + Temp + Temp:Var	6470951669	4196199910	116467086	0
Var	6518247622	4230464961	163763039	0
Temp + Var	6590587951	4276767932	236103368	0
Temp + Var + Gen	6605700412	4285507555	251215829	0
Temp + Temp:Var + Sign	6610511656	4288613918	256027073	0
Temp + Sign	6616766453	4292732031	262281870	0
Var + Gen	6654040956	4318228947	299556373	0
Var + Sign	6663301132	4325674689	308816549	0
Var + Temp + Temp:Var	6687473314	4340281054	332988731	0
Var + Temp + Temp:Var + Gen	6691804412	4341784043	337319829	0
Temp + Var + Sign	6770683578	4394652256	416198995	0
Temp + Var + Temp:Var + Sign	6826309994	4430331707	471825411	0

Table S12: Model selection table for the multi-point response to thermal selection analysis. Explanatory variables are absolute relative assay temperature (abbreviated as 'Temp' in the table), Type of genetic variation ('Var') and 'Relative selection temperature' (higher, lower or equal compared to the control treatment; 'Sign'). In the table '+' indicates an additive effect and ':' an interactive effect. We report WAIC, the standard error of WAIC ('SE'), the WAIC different to the bets model (dWAIC) as well as the WAIC weights.

**Model**	**WAIC**	**SE**	**dWAIC**	**weight**
Temp + $Temp^2$ + $Temp^3$ + Var + Sign	5.202676e+12	2.676116e+12	0	1
Temp + $Temp^2$ + $Temp^3$ + Sign	5.224529e+12	2.687450e+12	2.185324e+10	0
Temp + $Temp^2$ + $Temp^3$	5.290472e+12	2.721611e+12	8.779641e+10	0
Temp + $Temp^2$ + $Temp^3$ + Var	5.305165e+12	2.728286e+12	1.024890e+11	0
Temp + $Temp^2$ + Sign	5.621206e+12	2.893259e+12	4.185308e+11	0
Temp + Sign	5.653209e+12	2.909017e+12	4.505335e+11	0
Temp + $Temp^2$ + Var	5.683915e+12	2.926735e+12	4.812390e+11	0
Temp + Var + Sign	5.706087e+12	2.937974e+12	5.034112e+11	0
Temp + $Temp^2$ + Var + Sign	5.710847e+12	2.939725e+12	5.081711e+11	0
Temp + $Temp^2$	5.745498e+12	2.956929e+12	5.428220e+11	0
Temp	5.821055e+12	2.995173e+12	6.183796e+11	0
Temp + Var	5.888742e+12	3.031702e+12	6.860665e+11	0
Intercept	7.447238e+12	3.806424e+12	2.244562e+12	0
Var + Sign	7.528571e+12	3.847949e+12	2.325895e+12	0
Sign	7.543139e+12	3.856617e+12	2.340464e+12	0
Var	7.553221e+12	3.861681e+12	2.350545e+12	0

Table S13: Model selection table for the multi-point response to thermal selection analysis with additional explanatory variables including the number of generations (reduced data set). Explanatory variables are absolute relative assay temperature (abbreviated as 'Temp' in the table), Type of genetic variation ('Var'), 'Relative selection temperature' (higher, lower or equal compared to the control treatment; 'Sign') and the number of generations the experiment lasted ('Gen'; standardized). In the table '+' indicates an additive effect and ':' an interactive effect. We report WAIC, the standard error of WAIC ('SE'), the WAIC different to the bets model (dWAIC) as well as the WAIC weights.

**Model**	**WAIC**	**SE**	**dWAIC**	**weight**
Temp + $Temp^2$ + $Temp^3$ + Var + Sign	5.088572e+12	2.615790e+12	0	1
Temp + $Temp^2$ + $Temp^3$ + Var + Sign + Gen	5.115694e+12	2.627099e+12	2.712214e+10	0
Temp + $Temp^2$ + $Temp^3$	5.127109e+12	2.633056e+12	3.853778e+10	0
Temp + $Temp^2$ + $Temp^3$ + Sign	5.132144e+12	2.637492e+12	4.357262e+10	0
Temp + $Temp^2$ + $Temp^3$ + Sign + Gen	5.170180e+12	2.656259e+12	8.160809e+10	0
Temp + $Temp^2$ + $Temp^3$ + Var + Gen	5.208339e+12	2.676779e+12	1.197676e+11	0
Temp + $Temp^2$ + $Temp^3$ + Gen	5.218445e+12	2.680827e+12	1.298730e+11	0
Temp + $Temp^2$ + $Temp^3$ + Var	5.229531e+12	2.687119e+12	1.409589e+11	0
Temp + Sign	5.466533e+12	2.810131e+12	3.779613e+11	0
Temp + $Temp^2$ + Sign	5.487874e+12	2.821809e+12	3.993027e+11	0
Temp + $Temp^2$ + Var + Sign	5.506660e+12	2.831523e+12	4.180887e+11	0
Temp + $Temp^2$ + Gen	5.513372e+12	2.834466e+12	4.248003e+11	0
Temp + Var + Sign	5.519486e+12	2.838140e+12	4.309148e+11	0
Temp + Sign + Gen	5.524629e+12	2.841323e+12	4.360570e+11	0
Temp + Gen	5.544125e+12	2.850432e+12	4.555530e+11	0
Temp + $Temp^2$ + Sign + Gen	5.545664e+12	2.850498e+12	4.570923e+11	0
Temp	5.566529e+12	2.862299e+12	4.779578e+11	0
Temp + $Temp^2$ + Var	5.583444e+12	2.869222e+12	4.948724e+11	0
Temp + Var + Sign + Gen	5.595266e+12	2.875681e+12	5.066942e+11	0
Temp + Var + Gen	5.596104e+12	2.877852e+12	5.075325e+11	0
Temp + Var	5.611074e+12	2.886170e+12	5.225020e+11	0
Temp + $Temp^2$ + Var + Sign + Gen	5.617917e+12	2.886741e+12	5.293456e+11	0
Temp + $Temp^2$	5.621897e+12	2.888791e+12	5.333249e+11	0
Temp + $Temp^2$ + Var + Gen	5.623710e+12	2.890134e+12	5.351387e+11	0
Var	7.316852e+12	3.736721e+12	2.228280e+12	0
Sign	7.349525e+12	3.752395e+12	2.260953e+12	0
Intercept	7.365263e+12	3.758234e+12	2.276691e+12	0
Gen	7.393232e+12	3.774242e+12	2.304660e+12	0
Var + Sign	7.438562e+12	3.797575e+12	2.349991e+12	0
Sign + Gen	7.445837e+12	3.801358e+12	2.357266e+12	0
Var + Gen	7.488237e+12	3.823640e+12	2.399665e+12	0
Var + Sign + Gen	7.598070e+12	3.880620e+12	2.509498e+12	0

Table S14: Model selection table for the "hotter is better" analysis. Explanatory variables are 'Type of genetic variation' (standing genetic variation vs. de novo variation based on mutations) and 'Relative selection temperature' (higher, lower or equal compared to the control treatment). In the table '+' indicates an additive effect. We report WAIC, the standard error of WAIC ('SE'), the WAIC different to the bets model (dWAIC) as well as the WAIC weights.

**Model**	**WAIC**	**SE**	**dWAIC**	**weight**
Intercept	8660144	4963707	0	1
Sign	9131870	5239167	471726.2	0
Var	10781844	6203460	2121700.4	0
Var + Sign	10785915	6205959	2125770.9	0

Table S15: Model selection table for the "hotter is better" analysis using the reduced data set to included number of generations in the analysis. Explanatory variables are 'Type of genetic variation' (standing genetic variation vs. de novo variation based on mutations), 'Relative selection temperature' (higher, lower or equal compared to the control treatment) and the number of generations the experiment lasted ('Gen'; standardized). In the table '+' indicates an additive effect and ':' an interactive effect. We report WAIC, the standard error of WAIC ('SE'), the WAIC different to the bets model (dWAIC) as well as the WAIC weights.

**Model**	**WAIC**	**SE**	**dWAIC**	**weight**
Intercept	9814054	5627564	0	1
Sign	10399067	5969854	585012.6	0
Var	12218321	7032109	2404267.5	0
Var + Sign	12414274	7146141	2600220.1	0
Gen	14198806	8196894	4384751.6	0
Gen + Sign	14539062	8395046	4725008.6	0
Gen + Gen:Var	15499399	8957579	5685344.6	0
Gen + Gen:Var + Sign	15659298	9053435	5845243.5	0
Var + Gen	15703762	9078577	5889707.6	0
Var + Gen + Sign	15727338	9093349	5913283.9	0
Var + Gen + Var:Gen	16018136	9262200	6204082.3	0
Var + Gen + Var:Gen + Sign	16169460	9349567	6355405.5	0

Supplementary References

Aranguren-Gassis, M., Kremer, C., Klausmeier, C. & Litchman, E. 2019. Nitrogen limitation inhibits marine diatom adaptation to high temperatures. *Ecol. Lett.* **22**: 1860–1869.

Barton, S., Jenkins, J., Buckling, A., Schaum, C., Smirnoff, N., Raven, J. & Yvon-Durocher, G. 2020. Evolutionary temperature compensation of carbon fixation in marine phytoplankton. *Ecol. Lett.* **23**: 722–733.

Batarseh, T., Hug, S., Batarseh, S. & Gaut, B. 2020. Genetic mutations that drive evolutionary rescue to lethal temperature in *Escherichia coli*. *Genome Biol. Evol.* **12**: 2029–2044.

Bennett, A., Lenski, R. & Mittler, J. 1992. Evolutionary adaptation to temperature. I. Fitness responses of *Escherichia coli* to changes in its thermal environment. *Evolution* **46**: 16–30.

Bennett, A.F. & Lenski, R.E. 2007. An experimental test of evolutionary trade-offs during temperature adaptation. *Proc. Natl. Acad. Sci. U. S. A.* **104**: 8649–8654.

Berger, D., Walters, R.J. & Blanckenhorn, W.U. 2014. Experimental evolution for generalists and specialists reveals multivariate genetic constraints on thermal reaction norms. *J. Evol. Biol.* **27**: 1975–1989.

Breckels, R., Garner, S. & Neff, B. 2014. Rapid evolution in response to increased temperature maintains population viability despite genetic erosion in a tropical ectotherm. *Evol. Ecol.* **28**: 141–155.

Chakravarti, L.J., Beltran, V.H. & van Oppen, M.J.H. 2017. Rapid thermal adaptation in photosymbionts of reef-building corals. *Glob. Change Biol.* **23**: 4675–4688.

Chevin, L.M. 2011. On measuring selection in experimental evolution. *Biol. Lett.* **7**: 210–213.

Condon, C., Cooper, B.S., Yeaman, S. & Angilletta, M.J. 2014. Temporal variation favors the evolution of generalists in experimental populations of *Drosophila melanogaster*. *Evolution* **68**: 720–728.

Cooper, V.S., Bennett, A.F. & Lenski, R.E. 2001. Evolution of thermal dependence of growth rate of *Escherichia coli* populations during 20,000 generations in a constant environment. *Evolution* **55**: 889–896.

Cullum, A.J., Bennett, A.F. & Lenski, R.E. 2001. Evolutionary adaptation to temperature. IX. Preadaptation to novel stressful environments of *Escherichia coli* adapted to high temperature. *Evolution* **55**: 2194–2202.

Dam, H., deMayo, J., Park, G., Norton, L., He, X., Finiguerra, M., Baumann, H., Brennan, R. & Pespeni, M. 2021. Rapid, but limited, zooplankton adaptation to simultaneous warming and acidification. *Nat. Clim. Change* **11**: 780–786.

Duncan, A.B., Fellous, S., Quillery, E. & Kaltz, O. 2011. Adaptation of *Paramecium caudatum* to variable conditions of temperature stress. *Res. Microbiol.* **162**: 939–944.

Fragata, I., Lopes-Cunha, M., Barbaro, M., Kellen, B., Lima, M., Faria, G.S., Seabra, S.G., Santos, M., Simoes, P. & Matos, M. 2016. Keeping your options open: Maintenance of thermal plasticity during adaptation to a stable environment. *Evolution* **70**: 195–206.

Frenck, G., van der Linden, L., Mikkelsen, T.N., Brix, H. & Jorgensen, R.B. 2013. Response to multi-generational selection under elevated [CO2] in two temperature regimes suggests enhanced carbon assimilation and increased reproductive output in *Brassica napus* L. *Ecol. Evol.* **3**: 1163–1172.

Gilchrist, G., Huey, R. & Partridge, L. 1997. Thermal Sensitivity of *Drosophila melanogaster:* Evolutionary Responses of Adults and Eggs to Laboratory Natural Selection at Different Temperatures. *Physiol. Zool.* **70**: 403–414.

Gomez-Llano, M., Scott, E. & Svensson, E. 2021. The importance of pre- and postcopulatory sexual selection promoting adaptation to increasing temperatures. *Curr. Zool.* **67**: 321–327.

Hallsson, L.R. & Björklund, M. 2012. Selection in a fluctuating environment leads to decreased genetic variation and facilitates the evolution of phenotypic plasticity. *J. Evol. Biol.* **25**: 1275–1290.

Killeen, J., Gougat-Barbera, C., Krenek, S. & Kaltz, O. 2017. Evolutionary rescue and local adaptation under different rates of temperature increase: a combined analysis of changes in phenotype expression and genotype frequency in *Paramecium* microcosms. *Mol. Ecol.* **26**: 1734–1746.

Koch, E. & Guillaume, F. 2020. Restoring ancestral phenotypes is a general pattern in gene expression evolution during adaptation to new environments in *Tribolium castaneum*. *Mol. Ecol.* **29**: 3938–3953.

Lind, M., Zwoinska, M.K., Andersson, J., Carlsson, H., Krieg, T., Larva, T. & Maklakov, A.A. 2020. Environmental variation mediates the evolution of anticipatory parental effects. *Evol. Lett.* **4**: 371–381.

Listmann, L., LeRoch, M., Schlueter, L., Thomas, M.K. & Reusch, T.B.H. 2016. Swift thermal reaction norm evolution in a key marine phytoplankton species. *Evol. Appl.* **9**: 1156–1164.

Mongold, J., Bennett, A. & Lenski, R. 1999. Evolutionary adaptation to temperature. VII. Extension of the upper thermal limit of Escherichia coli. *Evolution* **53**: 386–394.

Mongold, J.A., Bennett, A.F. & Lenski, R.E. 1996. Evolutionary adaptation to temperature. IV. Adaptation of *Escherichia coli* at a niche boundary. *Evolution* **50**: 35–43.

O'Dea, R.E., Lagisz, M., Jennions, M.D., Koricheva, J., Noble, D.W., Parker, T.H., Gurevitch, J., Page, M.J., Stewart, G. & Nakagawa, D.M.S. 2021. Preferred reporting items for systematic reviews and meta-analyses in ecology andevolutionary biology: a PRISMA extension. *Biol. Rev.* **96**: 1695–1722.

O'Donnell, D.R., Hamman, C.R., Johnson, E.C., Kremer, C.T., Klausmeier, C.A. & Litchman, E. 2018. Rapid thermal adaptation in a marine diatom reveals constraints and trade-offs. *Glob. Change Biol.* **24**: 4554–4565.

Partridge, L., Barrie, B., Barton, N., Fowler, K. & French, V. 1995. Rapid Laboratory Evolution of Adult Life-History Traits in *Drosophila melanogaster* in Response to Temperature. *Evolution* **49**: 538–544.

Pfenninger, M. & Foucault, Q. 2020. Genomic processes underlying rapid adaptation of a natural *Chironomus riparius* population to unintendedly applied experimental selection pressures. *Mol. Ecol.* **29**: 536–548.

Plesnar-Bielak, A., Skrzynecka, A.M., Prokop, Z.M. & Radwan, J. 2012. Mating system affects population performance and extinction risk under environmental challenge. *Proc. R. Soc. B-Biol. Sci.* **279**: 4661–4667.

Rodriguez-Verdugo, A., Carrillo-Cisneros, D., Gonzalez-Gonzalez, A., Gaut, B.S. & Bennett, A.F. 2014. Different tradeoffs result from alternate genetic adaptations to a common environment. *Proc. Natl. Acad. Sci. U. S. A.* **111**: 12121–12126.

Romero-Olivares, A., Taylor, J. & Treseder, K. 2015. *Neurospora discreta* as a model to assess adaptation of soil fungi to warming. *BMC Evol. Biol.* **15**.

Rouco, M., Lopez-Rodas, V., Flores-Moya, A. & Costas, E. 2011. Evolutionary Changes in Growth Rate and Toxin Production in the Cyanobacterium *Microcystis aeruginosa* Under a Scenario of Eutrophication and Temperature Increase. *Microb. Ecol.* **62**: 265–273.

Sandberg, T.E., Pedersen, M., LaCroix, R.A., Ebrahim, A., Bonde, M., Herrgard, M.J., Palsson, B.O., Sommer, M. & Feist, A.M. 2014. Evolution of *Escherichia coli* to 42 degrees C and Subsequent Genetic Engineering Reveals Adaptive Mechanisms and Novel Mutations. *Mol. Biol. Evol.* **31**: 2647–2662.

Santos, M. 2007. Evolution of total net fitness in thermal lines: *Drosophila subobscura* likes it 'warm'. *J. Evol. Biol.* **20**: 2361–2370.

Santos, M., Brites, D. & Laayouni, H. 2006. Thermal evolution of pre-adult life history traits, geometric size and shape, and developmental stability in *Drosophila subobscura*. *J. Evol. Biol.* **19**: 2006–2021.

Schaum, C., Buckling, A., Smirnoff, N., Studholme, D. & Yvon-Durocher, G. 2018. Environmental fluctuations accelerate molecular evolution of thermal tolerance in a marine diatom. *Nat. Commun.* **9**: 1719.

Schaum, C.E., Barton, S., Bestion, E., Buckling, A., Garcia-Carreras, B., Lopez, P., Lowe, C., Pawar, S., Smirnoff, N., Trimmer, M. & Yvon-Durocher, G. 2017. Adaptation of phytoplankton to a decade of experimental warming linked to increased photosynthesis. *Nat. Ecol. Evol.* **1**: 0094.

Schluter, L., Lohbeck, K., Gutowska, M., Groger, J., Riebesell, U. & Reusch, T. 2014. Adaptation of a globally important coccolithophore to ocean warming and acidification. *Nat. Clim. Change* **4**: 1024–1030.

Shi, B.H. & Xia, X.H. 2003. Changes in growth parameters of *Pseudomonas pseudoalcaligenes* after ten months culturing at increasing temperature. *FEMS Microbiol. Ecol.* **45**: 127–134.

Sterne, J.A.C. & Egger, M. 2001. Funnel plots for detecting bias in meta-analysis: Guidelines on choice of axis. *J. Clin. Epidemiol.* **54**: 1046–1055.

Sterne, J.A.C., Egger, M. & Smith, G.D. 2001. Systematic reviews in health care: Investigating and dealing with publication and other biases in meta-analysis. *Br. Med. J.* **323**: 101–105.

Tarkington, J. & Zufall, R. 2021. Temperature affects the repeatability of evolution in the microbial eukaryote *Tetrahymena thermophila*. *Ecol. Evol.* **11**: 13139–13152.

Tseng, M., Bernhardt, J.R. & Chila, A.E. 2019. Species interactions mediate thermal evolution. *Evol. Appl.* **12**: 1463–1474.

Tseng, M. & O'Connor, M.I. 2015. Predators modify the evolutionary response of prey to temperature change. *Biol. Lett.* **11**: 20150798.

Van Doorslaer, W., Stoks, R., Duvivier, C., Bednarska, A. & De Meester, L. 2009. Population dynamics determine genetic adaptation to temperature in *Daphnia*. *Evolution* **63**: 1867–1878.

Van Doorslaer, W., Stoks, R., Jeppesen, E. & De Meester, L. 2007. Adaptive microevolutionary responses to simulated global warming in *Simocephalus vetulus*: a mesocosm study. *Glob. Change Biol.* **13**: 878–886.

Van Doorslaer, W., Stoks, R., Swillen, I., Feuchtmayr, H., Atkinson, D., Moss, B. & De Meester, L. 2010. Experimental thermal microevolution in community-embedded *Daphnia* populations. *Clim Res* **43**: 81–89.

Wu, E.J., Wang, Y.P., Yahuza, L., He, M.H., Sun, D.L., Huang, Y.M., Liu, Y.C., Yang, L.N., Zhu, W. & Zhan, J. 2020. Rapid adaptation of the Irish potato famine pathogen *Phytophthora infestans* to changing temperature. *Evol. Appl.* **13**: 768–780.

Xu, J. 2004. Genotype-environment interactions of spontaneous mutations for vegetative fitness in the human pathogenic fungus *Cryptococcus neoformans*. *Genetics* **168**: 1177–1188.

Zhang, C., Jansen, M., De Meester, L. & Stoks, R. 2019. Rapid evolution in response to warming does not affect the toxicity of a pollutant: Insights from experimental evolution in heated mesocosms. *Evol. Appl.* **12**: 977–988.
